# Mid-term clinical outcomes and complications of primary total knee arthroplasty in hemodialysis patients: a retrospective comparative cohort study

**DOI:** 10.1186/s12891-021-04810-8

**Published:** 2021-11-03

**Authors:** Sakumo Kii, Motoki Sonohata, Akira Hashimoto, Takema Nakashima, Atsushi Kawaguchi, Yosuke Matsumura, Takafumi Shimazaki, Satomi Nagamine, Masaaki Mawatari

**Affiliations:** 1 Department of Orthopaedic Surgery, Faculty of Medicine, Saga University, Nabeshima 5-1-1, Saga, 849-8501 Japan; 2 Education and Research Center for Community Medicine, Faculty of Medicine, Saga University, Nabeshima 5-1-1, Saga, 849-8501 Japan

**Keywords:** Hemodialysis, Total knee arthroplasty, Propensity score matching, American knee society score, Complications

## Abstract

**Background:**

Numerous patients who receive hemodialysis (HD) undergo total knee arthroplasty (TKA) due to advanced knee joint arthritis. However, there are few studies that describe the clinical outcomes and complications of TKA in HD patients. This study investigated the mid-term results of TKA in patients undergoing HD.

**Methods:**

This single-center retrospective study compared clinical and surgical outcomes following TKA in patients who were receiving HD with those who were not. We used propensity scores to match 21 knees of 18 patients who received HD to 706 knees of 569 patients who had not received HD, from a total of 727 knees (587 patients) that underwent primary unilateral TKA. The clinical outcomes were evaluated using the American Knee Society Score-knee (AKSS-knee) and AKSS-function scores. The primary surgical outcome measure was the number of knees with postoperative complications.

**Results:**

In both the HD and non-HD groups, postoperative AKSS-knee and function scores significantly improved when compared to preoperative values. Postoperative AKSS-knee and function scores were not significantly different between the groups. The number of knees with postoperative complications was larger in the HD group than the non-HD group within the first postoperative month, 0–12 months, 12–24 months, 0–24 months, and two years after surgery. Additionally, in the HD group, more complications occurred in the first month than any subsequent month in the two years after surgery.

**Conclusions:**

TKA improves AKSS-knee and function scores equivalently for HD patients and non-HD patients. However, HD patients develop more complications after TKA, especially within the first month. Therefore, surgeons who perform TKA for HD patients should obtain informed consent after explaining the possible complications, and HD patients should be carefully observed following TKA.

## Background

Chronic kidney failure is a widespread global disease. At the end of 2016, there were approximately 3.7 million patients with chronic kidney failure worldwide, with more than 70% receiving hemodialysis (HD) treatment [1]. Patients undergoing HD tend to have advanced osteoarthritis and osteonecrosis due to both aging and β2-microgloblin, which often necessitates total knee arthroplasty (TKA) [2–4]. For patients receiving HD, the relative risk of needing TKA is more than 2.0 compared with the general population [5]. TKA is a well-known surgery for knee joint pain and disorders related to arthritis, such as osteoarthritis, rheumatoid arthritis, and osteonecrosis [6, 7]. Numerous patients receiving HD undergo TKA due to advanced knee joint arthritis. However, there are few studies that describe the clinical outcomes and complications of TKA in patients undergoing HD. Here we describe mid-term outcomes and complications of primary TKA in patients receiving HD by using a propensity score matching method.

## Methods

This retrospective cohort study examined data obtained from the TKA database of our institution. The TKA database accumulates clinical data from patients who have undergone TKA, including the American Knee Society Score-knee (AKSS-knee) scores, AKSS-function scores, laboratory data, comorbidities, and postoperative complications. This was a single-center, retrospective study. The study protocol adhered to the ethical guidelines of the 1975 Declaration of Helsinki, and it was approved by the Institutional Review Board of our institution. All patients provided informed consent prior to participation in the study.

### Patients

**Fig. 1:**
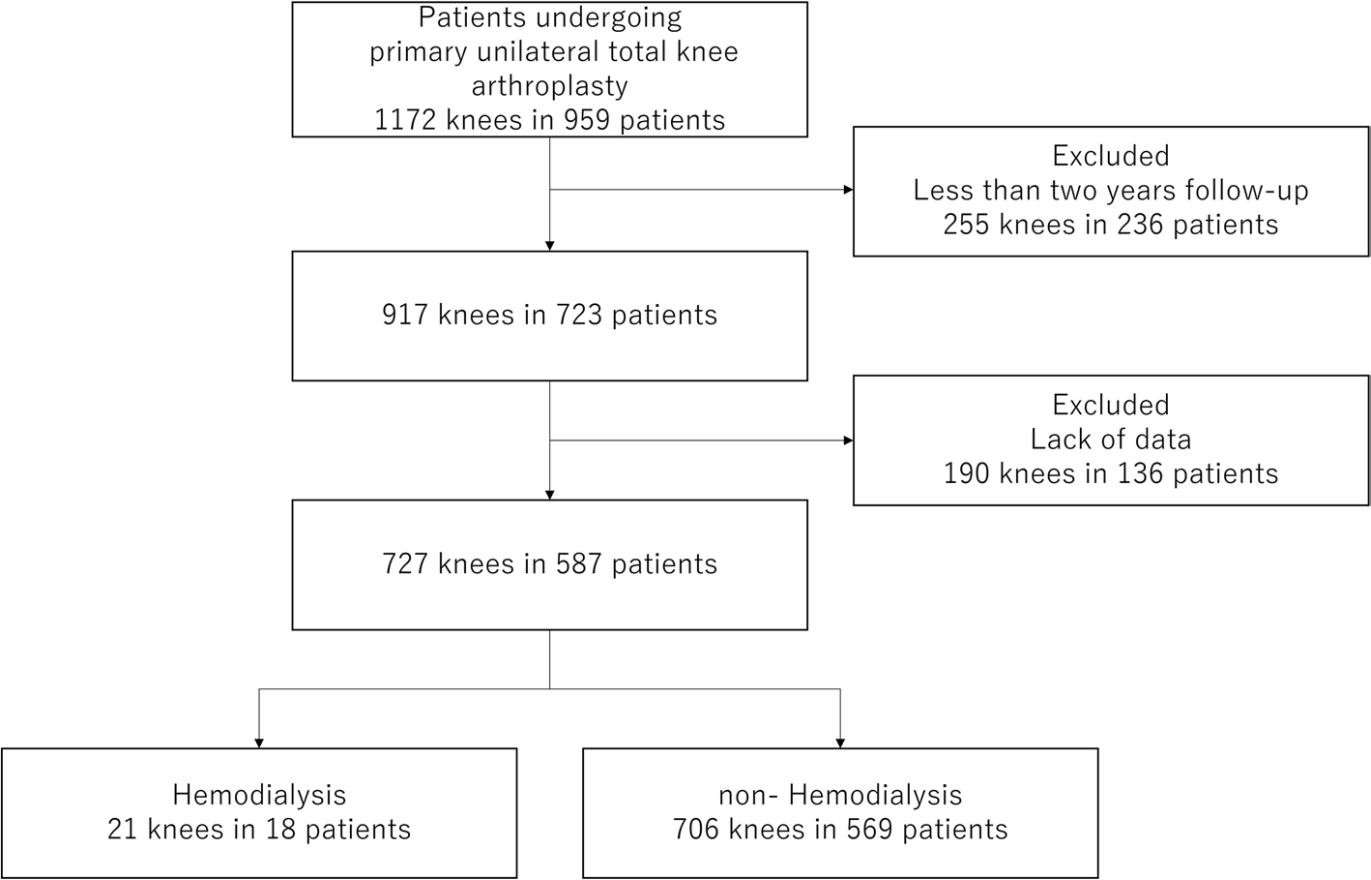
Flow chart depicting the study selection process and number of knees in the hemodialysis and non-hemodialysis patient groups undergoing primary unilateral total knee arthroplasty. HD: hemodialysis

First, data from 1172 knees (959 patients) that underwent primary unilateral TKA at our institution between January 2004 and August 2018 were extracted (Fig. 1). Next, we excluded 255 knees (236 patients) that had follow-up data spanning less than two years. Then, we excluded 190 knees (136 patients) with insufficient data. Finally, we divided the remaining 727 knees into two groups: an HD group (21 knees; 18 patients) and a non-HD group (706 knees; 569 patients). All patients in this study were Japanese.

### Operation

All TKA procedures were performed by senior surgeons using an air tourniquet and a medial parapatellar approach under spinal or general anesthesia, at the discretion of the anesthesiologist. The following types of implants were used: cruciate-retaining cemented implant (Vanguard Knee System [Zimmer Biomet, Indiana, USA]); posterior-stabilized cemented implant (Bi-Surface Total Knee System [Kyocera, Kyoto, Japan], NexGen LPS-Flex Knee [Zimmer Biomet, Indiana, USA], Persona [Zimmer Biomet, Indiana, USA], Scorpio NRG [Stryker, Michigan, USA], or Triathlon Total Knee System [Stryker, Michigan, USA]); or a posterior-stabilized cementless implant (Triathlon Total Knee System [Stryker, Michigan, USA]).

### Propensity score matching method

To minimize confounding, a propensity score matching method was used to match HD to non-HD patients. Using logistic regression, the propensity score was calculated from the following 25 variables: age, gender, body mass index (BMI), preoperative hemoglobin count, preoperative platelet count, preoperative albumin, preoperative AKSS-knee score, preoperative AKSS-function score, history of cancer, history of stroke, history of venous thromboembolism, osteoarthritis, rheumatoid arthritis, osteonecrosis, post-traumatic arthritis, post-osteotomy arthritis, diabetes mellitus, hypertension, cardiac disease, chronic respiratory disease, psychiatric disease, spinal disease, osteoporosis, chronic liver disease, and anemia, which was defined as less than 11.0 g of hemoglobin per deciliter of blood. These variables were selected based on prior studies [8–11].

Propensity matching was then performed, using nearest neighbor matching without replacement, with each HD patient matched to four control non-HD patients. A caliper width of 0.2 of the standard deviation of the logit of the propensity score was used. To check the balance of the matches, a standardized mean difference threshold of 0.1 was set as a reference.

### Outcomes

The primary outcome measures of this study were the postoperative AKSS-knee scores, postoperative AKSS-function scores, and number of knees with postoperative complications. The postoperative AKSS-knee and function scores were evaluated two years post-operation and at the patient’s last recorded follow-up visit. We also assessed subscores of the AKSS scales, including pain, total range of flexion, stability, flexion contracture, extension lag, walking, stairs, and walking aids used. The number of knees with postoperative complications, defined as any adverse event, was evaluated monthly for the first two years; complications were also grouped into time periods of 0–12 months, 12–24 months, and more than two years after TKA. Each type of complication that occurred during the observation period was recorded.

Secondary outcome measures included the postoperative length of hospital stay, postoperative follow-up period, operating time, anesthesia time, hemoglobin count on the first postoperative day and week, platelet count on the first postoperative day and week, and volume of allogenic blood transfusion.

### Statistical analyses

Statistical analyses were performed using JMP® Pro software (version 15.2.0, SAS Institute Japan Ltd., Tokyo, Japan). The categorical variables were expressed in absolute and percentage values, and the continuous variables were expressed as the mean ± standard deviation (SD). The Wilcoxon signed-rank test was used to compare the postoperative AKSS-knee and function scores with their respective preoperative scores within the same group. The Mann-Whitney U test was used to compare postoperative AKSS-knee scores, postoperative AKSS-function scores, postoperative length of hospital stay, postoperative follow-up period length, operating time, anesthesia time, hemoglobin count on the first postoperative day and in the first postoperative week, platelet count on the first postoperative day and week, and volume of allogenic blood transfusion between the HD and non-HD groups. Fisher’s exact test was used to compare the number of knees with postoperative complications and the presence of allogenic blood transfusion between the HD and non-HD groups. The Dunn test was used to compare the number of knees with postoperative complications in the HD group every month until two years post-operation. For all analyses, statistical significance was set at *p* <  0.05.

## Results

### Propensity score matching

**Fig. 2:**
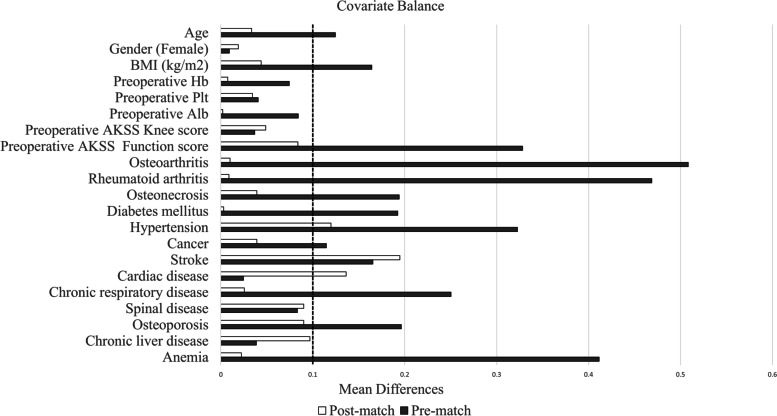
Covariate balance in the comparative study. The bar chart shows the changes in the standardized mean difference before (black) and after (white) matching. BMI: body mass index, Hb: hemoglobin, Plt: platelet, Alb: albumin, AKSS: American Knee Society Score

The propensity-matched population consisted of 21 knees (18 patients) in the HD group and 71 knees (70 patients) that were matched controls. Almost every variable achieved an appropriate balance, except hypertension, history of stroke, and cardiac disease. Baseline demographics for the matched study population are shown in Table 1 and Fig. 2.

**Table 1:** Baseline demographics for the propensity score matched study population

	Pre-match				Post-match			
1:4	HD (21 knees)	non-HD (706 knees)	p-value	Standardized difference	HD (21 knees)	non-HD (71 knees)	p-value	Standardized difference
Age	66.9 ± 9.3	73.4 ± 8.6	0.001	0.124	66.9 ± 9.3	68.5 ± 12.2	0.312	0.033
Gender (Female)	17 (81.0)	569 (80.6)	1.000	0.009	17 (81.0)	58 (81.7)	1.000	0.0189
BMI (kg/m^2^)	22.74 ± 4.85	25.78 ± 3.95	0.001	0.164	22.74 ± 4.85	23.48 ± 4.44	0.446	0.044
Preoperative Hb	11.88 ± 1.35	12.54 ± 1.42	0.004	0.074	11.88 ± 1.35	11.81 ± 1.45	0.849	0.007
Preoperative Plt	21.25 ± 6.05	21.90 ± 6.6	0.829	0.041	21.25 ± 6.05	21.80 ± 8.17	0.919	0.034
Preoperative Alb	3.74 ± 0.32	3.98 ± 0.38	0.002	0.084	3.74 ± 0.32	3.732 ± 0.48	0.685	0.002
Preoperative AKSS Knee score	44.9 ± 18.8	46.2 ± 16.0	0.760	0.037	44.9 ± 18.8	46.6 ± 16.7	0.787	0.049
Preoperative AKSS Function score	30.7 ± 20.9	41.5 ± 19.9	0.018	0.328	30.7 ± 20.9	33.0 ± 21.4	0.562	0.084
Osteoarthritis	14 (66.7)	617 (87.4)	0.014	0.508	14 (66.7)	47 (66.2)	1.000	0.010
Rheumatoid arthritis	6 (28.6)	74 (10.5)	0.021	0.469	6 (28.6)	20 (28.2)	1.000	0.009
Osteonecrosis	1 (4.8)	10 (1.4)	0.277	0.194	1 (4.8)	4 (5.6)	1.000	0.039
Post-traumatic arthritis	0 (0.0)	5 (0.7)	1.000	16.724	0 (0.0)	0 (0.0)	N/A	N/A
Post-osteotomy arthritis	0 (0.0)	4 (0.6)	1.000	18.678	0 (0.0)	0 (0.0)	N/A	N/A
Diabetes mellitus	5 (23.8)	114 (16.2)	0.366	0.192	5 (23.8)	17 (23.9)	1.000	0.003
Hypertension	8 (38.1)	381 (54.0)	0.184	0.323	8 (38.1)	23 (32.4)	0.612	0.120
Cancer	1 (4.8)	53 (7.5)	1.000	0.115	1 (4.8)	4 (5.6)	1.000	0.039
Stroke	1 (4.8)	63 (8.9)	1.000	0.165	1 (4.8)	1 (1.4)	0.406	0.195
Cardiac disease	3 (14.3)	107 (15.2)	1.000	0.025	3 (14.3)	7 (9.8)	0.690	0.136
Chronic respiratory disease	1 (4.8)	5 (0.7)	0.162	0.250	1 (4.8)	3 (4.2)	1.000	0.026
Psychiatric disease	0 (0.0)	19 (2.7)	1.000	8.506	0 (0.0)	0 (0.0)	N/A	N/A
Spinal disease	2 (9.5)	51 (7.2)	0.661	0.083	2 (9.5)	5 (7.0)	0.657	0.090
Osteoporosis	2 (9.5)	32 (4.5)	0.257	0.196	2 (9.5)	5 (7.0)	0.657	0.090
Venous thromboembolism	0 (0.0)	1 (0.1)	1.000	37.770	0 (0.0)	0 (0.0)	N/A	N/A
Chronic liver disease	1 (4.8)	28 (4.0)	0.580	0.039	1 (4.8)	5 (7.0)	1.000	0.097
Anemia	6 (28.6)	87 (12.3)	0.041	0.411	6 (28.6)	21 (29.6)	1.000	0.022

Data are presented as the mean ± SD or N(%). *HD* hemodialysis, *BMI* body mass index, *Hb* hemoglobin, *Plt* platelet, *Alb* albumin, *AKSS* American Knee Society Score, *N/A* not applicable

### Intragroup comparisons

In both the HD and non-HD groups, 2 years the postoperative AKSS-knee and function scores significantly improved compared to their preoperative values. However, the postoperative AKSS-knee and function scores obtained at the 2-year follow-up were not significantly different than those obtained at the last follow-up in either group (Table 2).

**Table 2:** Intragroup comparison of AKSS-knee and AKSS-function scores

	Pre-operation	Two year follow-up	Last follow-up	p-value^a^	p-value^b^	p-value^c^
HD group						
AKSS Knee Score	44.9 ± 18.8 (10–72)	95.0 ± 9.8 (72–100)	95.4 ± 7.7 (72–100)	< 0.0001	< 0.0001	0.7684
AKSS Function Score	30.7 ± 20.9 (0–70)	73.1 ± 27.2 (0–100)	66.9 ± 33.0 (0–100)	< 0.0001	< 0.0001	0.1567
non-HD group						
AKSS Knee Score	46.6 ± 16.7 (0–80)	96.8 ± 5.5 (69–100)	97.6 ± 3.4 (83–100)	< 0.0001	< 0.0001	0.2696
AKSS Function Score	33.0 ± 21.4 (0–70)	78.9 ± 18.8 (15–100)	79.2 ± 18.2 (15–100)	< 0.0001	< 0.0001	0.8616

p-value^a^: Pre-operation vs. Two year follow-up

*p*-value^b^: Pre-operation vs. Last follow-up

p-value^c^: Two year follow-up vs. Last follow-up

Continuous variables are expressed as the mean ± standard deviation (range). HD: hemodialysis, *AKSS* American Knee Society Score

### Comparison between groups

There were no significant differences between the HD group and non-HD group in the postoperative AKSS-knee and function scores that were obtained two years after surgery or at the last follow-up. There were no significant differences between the groups in the change in scores post-operation versus pre-operation. Among the individual items of the AKSS-knee and function scales, there were significant differences between the HD group and non-HD group in the AKSS-knee stability subscore at the last follow-up (24.5 ± 1.5 vs. 25.0 ± 0.0; *P* = 0.0089) and the AKSS-function walking subscore at the last follow-up (38.6 ± 16.5 vs. 46.1 ± 7.7; *P* = 0.033). The results for the primary and secondary outcome measures are shown in Table 3.

**Table 3:** Comparison of primary and secondary outcome measures between the HD and non-HD groups

	HD group	non-HD group	p-value
AKSS-knee score at the two year follow-up	95.0 ± 9.8 (72–100)	96.8 ± 5.5 (69–100)	0.4641
Pain	48.1 ± 6.0 (30–50)	49.2 ± 2.8 (30–50)	0.7978
Total range of flexion	23.8 ± 3.6 (12–25)	24.2 ± 1.9 (14–25)	0.3893
Stability	25.0 ± 0.0 (25–25)	25.0 ± 0.0 (25–25)	1.0000
Flexion contracture	1.2 ± 3.3 (0–15)	0.5 ± 0.9 (0–2)	0.7817
Extension lag	0.7 ± 1.8 (0–5)	0.5 ± 2.7 (0–20)	0.1175
Alignment	0.0 ± 0.0(0–0)	0.5 ± 2.3 (0–15)	0.2653
Increase from preoperative score	50.1 ± 19.3 (26–90)	50.2 ± 17.1 (14–91)	0.8633
AKSS-function score at the two year follow-up	73.1 ± 27.2 (0–100)	78.9 ± 18.8 (15–100)	0.6314
Walking	41.4 ± 14.2 (0–50)	45.8 ± 7.9 (20–50)	0.2368
Stairs	33.3 ± 12.8 (0–50)	36.0 ± 10.8 (0–50)	0.6052
Walking aids used	2.1 ± 4.6 (0–20)	3.0 ± 4.9 (0–20)	0.2639
Increase from preoperative score	42.4 ± 29.7 (− 35–90)	45.9 ± 20.6 (− 20–90)	0.8923
AKSS-knee score at the last follow-up	95.4 ± 7.7 (72–100)	97.6 ± 3.4 (83–100)	0.9048
Pain	48.8 ± 3.1 (40–50)	49.3 ± 1.8 (45–50)	0.8595
Total range of flexion	23.5 ± 3.6(12–25)	24.2 ± 2.4 (8–25)	0.8083
Stability	24.5 ± 1.5 (20–25)	25.0 ± 0.0 (25–25)	0.0089
Flexion contracture	1.2 ± 3.3 (0–15)	0.5 ± 1.0 (0–5)	0.6166
Extension lag	0.2 ± 1.1 (0–5)	0.07 ± 0.6 (0–5)	0.3572
Alignment	0.0 ± 0.0(0–0)	0.3 ± 1.5 (0–9)	0.3409
Increase from preoperative score	50.5 ± 18.5 (27–90)	51.0 ± 16.7 (20–98)	0.7979
AKSS-function score at the last follow-up	66.9 ± 33.0 (0–100)	79.2 ± 18.2(15–100)	0.2674
Walking	38.6 ± 16.5 (0–50)	46.1 ± 7.7 (20–50)	0.0330
Stairs	30.0 ± 16.1 (0–50)	36.1 ± 9.9 (0–50)	0.3110
Walking aids used	2.1 ± 4.6 (0–20)	3.0 ± 4.6 (0–20)	0.2640
Increase from preoperative score	36.2 ± 34.3 (− 35–90)	46.1 ± 20.0 (10–85)	0.3269
Postoperative length of stay (days)	25.4 ± 30.8 (9–154)	17.2 ± 3.4 (12–32)	0.4968
Postoperative follow-up period (months)	42.2 ± 25.2 (24–98)	62.7 ± 30.4 (25–150)	0.0047
Operating time (minutes)	83.8 ± 14.5 (64–109)	80.4 ± 20.6 (46–139)	0.2391
Anesthesia time (minutes)	120.3 ± 17.3 (90–155)	117.8 ± 28.1 (67–214)	0.3473
Hemoglobin count on the first postoperative day (g/dL)	10.4 ± 1.5 (7.8–13.2)	10.1 ± 1.2 (7.2–13.2)	0.4453
Hemoglobin count at one week postoperation (g/dL)	9.3 ± 1.2 (7–11.2)	9.7 ± 1.3 (6.8–14.1)	0.3147
Platelet count on the first postoperative day (× 10^9^/L)	18.2 ± 6.1 (8.4–31.3)	17.9 ± 6.8 (5.3–34.0)	0.8269
Platelet count at one week postoperation (× 10^9^/L)	23.2 ± 7.0 (9.1–38.1)	25.2 ± 8.6 (3.0–50.2)	0.2764
Volume of allogenic blood transfusion (mL)	1.1 ± 3.2 (0–14)	0.08 ± 0.5 (0–4)	0.0077

Continuous variables are expressed as the mean ± SD (range). *AKSS* American Knee Society Score, *HD* hemodialysis

**Fig. 3:**
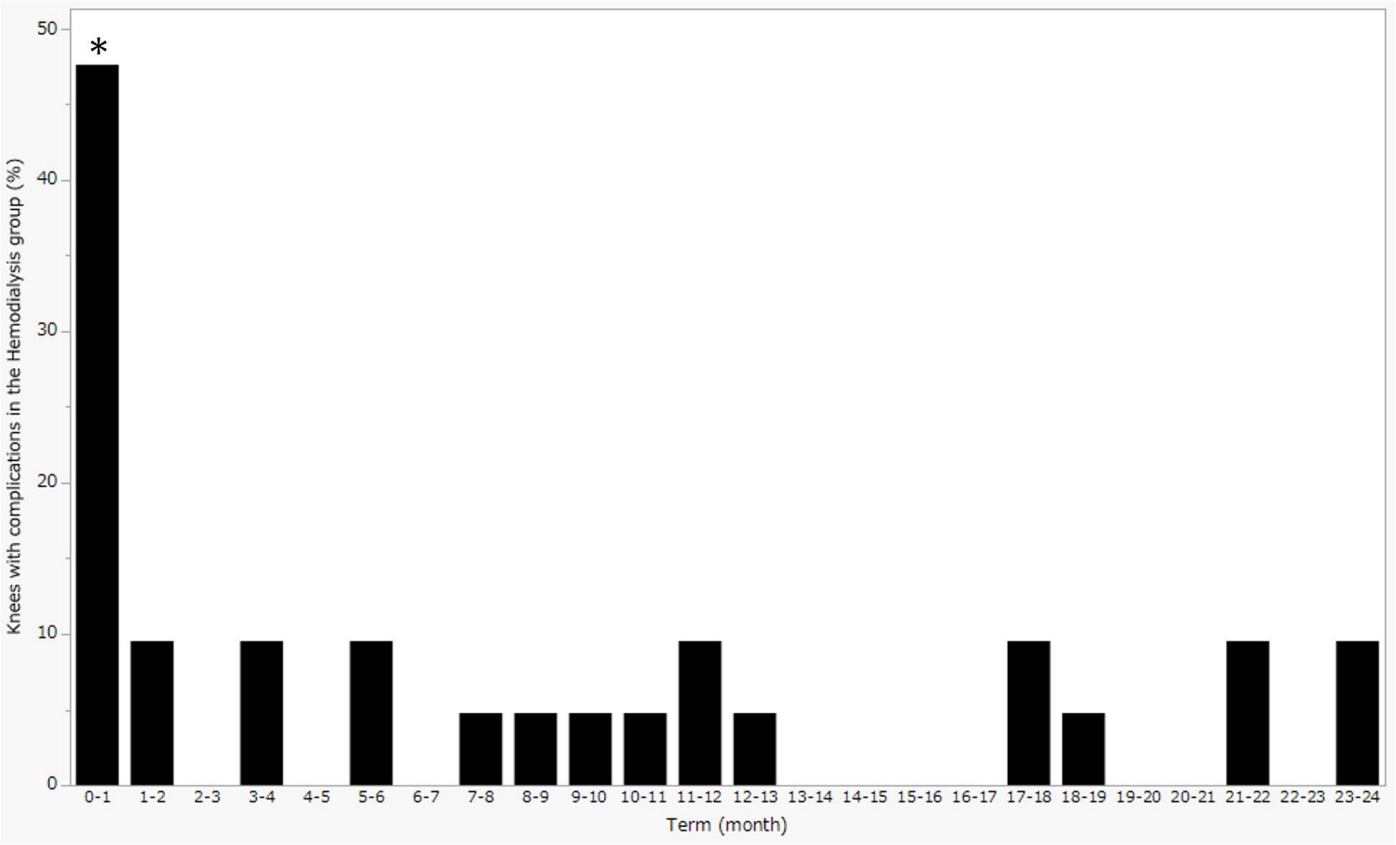
The number of knees with postoperative complications in the hemodialysis group at each month in the two years after surgery. ∗ denotes a significant difference between the 0–1 month period and the other time periods. *p* <  0.0001

The number of knees with postoperative complications was greater in the HD group than the non-HD group in the first postoperative month, 0–12 months, 12–24 months, 0–24 months, and more than two years after surgery (Table 4). Additionally, in the HD group, the number of knees with postoperative complications was larger in the first month than any subsequent month in the first two years after surgery (Fig. 3). When we examined each type of complication, we found that anemia, shunt failure, carpal tunnel syndrome, and severe aortic stenosis occurred more frequently in the HD group than the non-HD group (Table 5).

**Table 4:** Number of knees with postoperative complications in the HD and non-HD groups

	HD group	non-HD group	p-value	Odds ratio	95% CI
Any complications within the time period					
0–12 months	13 (61.9)	20 (28.2)	0.0085	4.14	1.49–11.51
12–24 months	7 (33.3)	7 (9.9)	0.0149	4.57	1.38–15.13
0–1 month	10 (47.6)	12 (16.9)	0.0074	4.47	1.55–12.87
1–2 months	2 (9.5)	1 (1.4)	0.1293	7.37	0.63–85.68
2–3 months	0 (0.0)	0 (0.0)	N/A	N/A	N/A
3–4 months	2 (9.5)	2 (2.8)	0.2227	3.63	0.48–27.50
4–5 months	0 (0.0)	0 (0.0)	N/A	N/A	N/A
5–6 months	2 (9.5)	1 (1.4)	0.1293	7.37	0.63–85.68
6–7 months	0 (0.0)	0 (0.0)	N/A	N/A	N/A
7–8 months	1 (4.8)	2 (2.8)	0.5449	1.73	0.15–20.02
8–9 months	1 (4.8)	0 (0.0)	0.2283	N/A	N/A
9–10 months	1 (4.8)	0 (0.0)	0.2283	N/A	N/A
10–11 months	1 (4.8)	1 (1.4)	0.4064	3.50	0.21–58.48
11–12 months	2 (9.5)	4 (5.6)	0.6166	1.76	0.30–10.37
12–13 months	1 (4.8)	0 (0.0)	0.2283	N/A	N/A
13–14 months	0 (0.0)	1 (1.4)	1.0000	N/A	N/A
14–15 months	0 (0.0)	0 (0.0)	N/A	N/A	N/A
15–16 months	0 (0.0)	1 (1.4)	1.0000	N/A	N/A
16–17 months	0 (0.0)	1 (1.4)	1.0000	N/A	N/A
17–18 months	2 (9.5)	0 (0.0)	0.0502	N/A	N/A
18–19 months	1 (4.8)	0 (0.0)	0.2283	N/A	N/A
19–20 months	0 (0.0)	0 (0.0)	N/A	N/A	N/A
20–21 months	0 (0.0)	0 (0.0)	N/A	N/A	N/A
21–22 months	2 (9.5)	2 (2.8)	0.2227	3.63	0.48–27.50
22–23 months	0 (0.0)	1 (1.4)	1.0000	N/A	N/A
23–24 months	2 (9.5)	2 (2.8)	0.2227	3.63	0.48–27.50
0–24 months	13 (61.9)	25 (35.2)	0.0429	2.99	1.09–8.18
Beyond two years	12 (57.1)	21 (29.6)	0.0364	3.17	1.16–8.66

Categorical variables are expressed as the number of knees (%). *HD* hemodialysis, *N/A* not applicable, *CI* confidence interval

**Table 5:** Comparison of complications between the HD and non-HD groups during the entire observation period

	HD group	non-HD group	p-value	Odds ratio	95% CI
Anemia requiring allogenic blood transfusion	4 (19.1)	2 (2.8)	0.0230	8.12	1.37–48.06
Shunt failure	6 (28.6)	0 (0.0)	< 0.0001	N/A	N/A
Carpal tunnel syndrome	3 (14.3)	0 (0.0)	0.0106	N/A	N/A
Severe aortic stenosis	3 (14.3)	0 (0.0)	0.0106	N/A	N/A
Periprosthetic joint infection	1 (4.8)	3 (4.2)	1.0000	0.88	0.09–8.96
Ileus	2 (9.5)	0 (0.0)	0.0502	N/A	N/A
Pressure ulcer	3 (14.3)	2 (2.8)	0.0763	0.17	0.03–1.12
Hypoglycemia	2 (9.5)	0 (0.0)	0.0502	N/A	N/A
Hematoma around the knee	2 (9.5)	2 (2.8)	0.2227	0.28	0.04–2.09
Shoulder osteoarthritis	1 (4.8)	0 (0.0)	0.2283	N/A	N/A
Pneumomycosis	2 (9.5)	0 (0.0)	0.0502	N/A	N/A
Vertebral compression fracture	2 (9.5)	0 (0.0)	0.0502	N/A	N/A
Erythema induratum of Bazin	2 (9.5)	0 (0.0)	0.0502	N/A	N/A
Femoral neck fracture	1 (4.8)	0 (0.0)	0.2283	N/A	N/A
Acute cholecystitis	1 (4.8)	0 (0.0)	0.2283	N/A	N/A
Intracardiac thrombus	1 (4.8)	0 (0.0)	0.2283	N/A	N/A
Infective endocarditis	1 (4.8)	0 (0.0)	0.2283	N/A	N/A
Lumber spinal stenosis	2 (9.5)	1 (1.4)	0.1293	0.14	0.01–1.58
Transient ischemic attack	2 (9.5)	1 (1.4)	0.1293	0.14	0.01–1.58
Subconjunctival hemorrhage	1 (4.8)	0 (0.0)	0.2283	N/A	N/A
Posterior interosseous nerve palsy	1 (4.8)	0 (0.0)	0.2283	N/A	N/A
Cervical spondylotic myelopathy	2 (9.5)	0 (0.0)	0.0502	N/A	N/A
Sudden cardiac arrest	1 (4.8)	0 (0.0)	0.2283	N/A	N/A
Death	2 (9.5)	1 (1.4)	0.1293	0.14	0.01–1.58
Distal radial fracture	1 (4.8)	0 (0.0)	0.2283	N/A	N/A
Colon carcinoma in adenoma	1 (4.8)	0 (0.0)	0.2283	N/A	N/A
Acute myocardial infarction	1 (4.8)	1 (1.4)	0.4064	0.29	0.02–4.77
Urinary tract infection	2 (9.5)	0 (0.0)	0.0502	N/A	N/A
Sepsis	1 (4.8)	0 (0.0)	0.2283	N/A	N/A
Pneumonia	1 (4.8)	2 (2.8)	0.5449	0.58	0.05–6.73
Ascending colonic diverticular bleeding	1 (4.8)	0 (0.0)	0.2283	N/A	N/A
Gastric ulcer	1 (4.8)	1 (1.4)	0.4064	0.29	0.02–4.77

Categorical variables are expressed as the number of knees (%). HD: hemodialysis, N/A: not applicable, CI: confidence interval

## Discussion

This study highlights two important clinical findings. First, TKA for HD patients improves their AKSS-knee and function scores, with no significant differences compared to patients who do not receive HD. Second, HD patients are more likely to develop complications after TKA than non-HD patients, especially within the first month after TKA.

Previous studies of TKA for HD patients were often simple comparisons. However, simple comparisons are difficult because the demographic and clinical characteristics of HD patients differ greatly from that of non-HD patients. In the current study, we matched demographic and clinical factors to permit a more precise analysis of TKA for HD patients by using a propensity score matching method.

Utrilla et al. [12] reported that the preoperative and postoperative AKSS-knee scores were not significantly different between patients with or without end-stage renal disease (ESRD). However, they found that the preoperative and postoperative AKSS-function scores were significantly lower in the ESRD group than the non-ESRD group. Interestingly, there was no significant difference between groups in the mean gain in AKSS-function (45.1 vs. 43.2). In the current study, the postoperative AKSS-function scores were not significantly different between the HD and non-HD groups. This may be because the propensity score matching method included the preoperative AKKS-function scores. Based on the above results, we conclude that TKA is beneficial for patients undergoing HD. However, when we examined the individual items of the AKSS-knee and function scales, the AKSS-knee stability and AKSS-function walking scores were significantly lower in the HD group at the last follow-up, compared to the non-HD group.

Regarding instability in HD patients, Malkani et al. [13] reported that impaired musculo-skeletal function and decreased muscular tone associated with renal osteodystrophy led to increased joint laxity and subsequent dislocation in dialysis patients who underwent total hip arthroplasty. In a group of HD patients undergoing TKA, Lo et al. [14] reported one case of knee instability that required casting and had a poor final functional outcome. In our study, we believe that HD patients have diminished knee stability compared with non-HD patients due to decreased muscle tone and muscle weakness. Fortunately, there were no cases of knee dislocation or instability requiring casting in our study. However, a strict approach for appropriate implant selection, component placement and preservation of the soft tissue structure should be maintained to prevent knee instability following TKA in HD patients.

The prevalence of fatigue in patients receiving renal replacement therapy ranges from 60% to as high as 97%, which is higher than the incidence of fatigue in patients who are not undergoing renal replacement therapy, owing to physiological, sociodemographic, psychological, behavioral, and dialysis-related factors [15]. In our study, fatigue may be the reason why the walking subscore of the AKSS-function scale at the last follow-up was lower in the HD group than the non-HD group. Low levels of physical activity are associated with an increased risk for mortality among dialysis patients [16]. Therefore, exercise therapy aimed to preserve or enhance physical activity should be considered for HD patients after TKA.

HD patients developed more complications after TKA than non-HD patients, especially within the first month. This may be due to predisposing conditions, such as a diminished blood supply, compromised immune system, amyloidosis, poor bone quality, electrolyte abnormalities, and impaired wound healing [9, 17, 18]. In addition, operative stress may contribute to the occurrence of complications in the first month. In the HD group, 10 out of 21 knees (47.6%) experienced adverse events in the first postoperative month, which is considered the perioperative period. The odds ratio for complications in this period was 4.47 (95% confidence interval [CI], 1.55–12.87; *P* = 0.0074) when comparing the HD and non-HD groups. Similar to our study, Ottesen et al. [19] reported that dialysis-dependent patients were 2.01 times more likely to have any adverse events within 30 days of TKA. Therefore, HD patients should be carefully observed in the first month after TKA.

Additionally, Ottesen et al. [19] reported that dialysis-dependent patients were 6.71 times more likely to die within 30 days of TKA. In our study, no patients died within 30 days of TKA. However, two patients with HD died during the follow-up period. One patient died of cardiac arrest during HD 81 months after TKA at the age of 76. She had received HD for 285 months, beginning when she was 53 years old. The other patient died of septic shock 57 months after TKA at the age of 72; she had received HD for 69 months, beginning when she was 66 years old. Generally, the life expectancy of HD patients is less than half that of the general population [20]. It was not clear whether TKA affected the life expectancy of the HD patients in our study, because a long time elapsed between TKA and death in our two patients.

When we examined each type of complication, we found that anemia, shunt failure, carpal tunnel syndrome, and severe aortic stenosis occurred more frequently in the HD group than the non-HD group.

Anemia that required allogenic blood transfusion occurred in four knees (19.1%) in the HD group during the observation period, which was more frequent than in the non-HD group (2 knees [2.8%]; *P* = 0.0230). However, there were no significant differences between the groups in the hemoglobin count on the first postoperative day or in the first postoperative week. A possible reason for this result is that surgeons may have decided to utilize allogenic blood transfusion for HD patients earlier due to poor tolerance for volume load and loss [17]. Dialysis patients have a potential for hemorrhage due to the destruction of platelets by heparin during dialysis [21]. To avoid heparization, which can lead to bleeding at the surgical site, dialysis within 24 h post-operation is not recommended [21]. Therefore, adjustment of the TKA and HD schedules may enable patients to avoid allogenic blood transfusion.

Shunt failure is one of the most important complications of HD. Al-Jaishi et al. [22] reported that the primary patency rate of an arteriovenous fistula was 60% at one year and 51% at two years. As their report suggested, shunt failure was a common complication, and its frequency increased with age. Sridharan et al. [23] reported that HD access satisfaction is associated with better health-related quality of life. In our study, shunt failure occurred 6 times (6 knees in 6 patients; 28.6%) in the HD group across the whole observation period. Therefore, to improve health-related quality of life, surgeons should work closely with the dialysis care team, including the nephrologist and vascular surgeon.

Carpal tunnel syndrome is a well-known complication of HD that is caused by amyloid deposition [24]. In our study, carpal tunnel syndrome was reported 3 times in the HD group (3 knees in 3 patients; 14.3%) in the HD group during the observation period, while it did not occur in the non-HD group. Filho et al. [25] reported that patients with carpal tunnel syndrome have a high prevalence of anxiety (28.7%) and depression (37.6%). Associations between TKA, carpal tunnel syndrome, anxiety, and depression have not been explored. However, anxiety and depression are risk factors for postoperative pain-related symptoms and complications in patients undergoing primary TKA [26]. Therefore, surgeons should be aware that HD patients undergoing primary TKA may be at a greater risk for carpal tunnel syndrome, anxiety, and depression.

Aortic stenosis is another serious complication in HD patients. Several studies reported that aortic stenosis tends to occur more frequently in HD patients than the general population, due to the mechanisms of vascular calcification that are accelerated by the rise of serum calcium and phosphorus concentrations [27–29]. Taniguchi et al. [30] reported that the hazard ratios of sudden death in asymptomatic and symptomatic HD patients with severe aortic stenosis were 7.79 and 3.87, respectively. In our study, severe aortic stenosis occurred following TKA in three knees (14.3%) in the HD group during the observation period, while it did not occur in the non-HD group. Therefore, when performing TKA for HD patients, preoperative screening for aortic stenosis is important to avoid sudden death. Additionally, if the surgeon treats osteoporosis with medications such as active vitamin D preparations to prevent a periprosthetic knee fracture, the serum calcium and phosphorus concentrations should be monitored carefully to prevent aortic valve calcification [31].

Periprosthetic joint infection (PJI) is one of the most important complications in patients undergoing TKA. Lee et al. [5] reported that patients with ESRD experienced PJI more frequently than those without ESRD. In our study, PJI occurred in one knee (4.8%) in the HD group during the observation period. There were no significant differences in PJI occurrence between the HD and non-HD groups. However, in this case, the patient suffered from recurrent PJI that resulted in a long, 5-month hospitalization. Lee et al. [5] also reported that the length of hospital stay of ESRD patients was significantly longer than that of non-ESRD patients when there were complications, including PJI. Therefore, surgeons should pay careful attention to the symptoms of PJI in HD patients to enable early detection and treatment.

Given the complications described above, informed consent should be obtained from HD patients before TKA, after the possible complications associated with HD and the influence of operative stress have been thoroughly discussed.

This study has four limitations. First, this study used a retrospective single-center design, with a relatively small patient sample. However, we used a propensity score matching method, which we believe minimized confounding. Studies with a larger number of patients are needed in the future to correct the covariate balance for variables such as hypertension, history of stroke, and cardiac disease, which were not fully adjusted for in this study. Second, this study included mid-term results. Studies with a long-term follow-up are needed in the future. Third, we performed TKA with various types of implants; a unified implant design may produce different results. Hence, a study of TKA using a unified type of implant may be needed in the future. Finally, all patients in this study were Japanese. The prevalence of HD is higher and the hazard ratio is lower in Japanese patients compared to patients in other countries [32, 33]. Therefore, different results may be obtained in other populations.

## Conclusions

TKA improved the AKSS-knee and function scores to the same extent in HD patients as in non-HD patients. However, HD patients developed more complications after TKA, especially within the first month. Therefore, surgeons who perform TKA for HD patients should obtain informed consent for surgery after explaining the possible complications, and HD patients should be carefully observed following TKA.

## Data Availability

The datasets analyzed during the current study are available from the corresponding author on reasonable request.

## Abbreviations

AKSS-function: American Knee Society Score-function
AKSS-knee: American Knee Society Score-knee
BMI: Body mass index
ESRD: End-stage renal disease
HD: Hemodialysis
PJI: Periprosthetic joint infection
SD: Standard deviation
TKA: Total knee arthroplasty

## References

[1] Fresenius Medical Care. Annual report 2016, chapter 2. https://www.freseniusmedicalcare.com/fileadmin/data/com/pdf/investors/News___Publications/Annual_Reports/2016/FMC_AnnualReport_2016_en.pdf. Accessed 4 August 2021.

[2] Ponnusamy KE, Jain A, Thakkar SC, Sterling RS, Skolasky RL, Khanuja HS (2015). Inpatient mortality and morbidity for dialysis-dependent patients undergoing primary total hip or knee arthroplasty. J Bone Joint Surg Am.

[3] Jevtic V (2003). Imaging of renal osteodystrophy. Eur J Radiol.

[4] Cobby MJ, Adler RS, Swartz R, Martel W (1991). Dialysis-related amyloid arthropathy: MR findings in four patients. AJR Am J Roentgenol.

[5] Lee SH, Lin YC, Chang CJ, Fan Chiang CY, Chen SY, Chang YH, Hsieh PH, Chang CH (2020). Outcome and cost analysis of primary total knee arthroplasty in end-stage renal disease patients: a nationwide population-based study. Biomed J.

[6] Coventry MB (1979). Two-part total knee arthroplasty: evolution and present status. Clin Orthop Relat Res.

[7] Robinson RP (2005). The early innovators of today's resurfacing condylar knees. J Arthroplast.

[8] Gould D, Dowsey M, Jo I, Choong P (2020). Patient-related risk factors for unplanned 30-day readmission following total knee arthroplasty: a narrative literature review. ANZ J Surg.

[9] Canton G, Ratti C, Fattori R, Hoxhaj B, Murena L (2017). Periprosthetic knee fractures. A review of epidemiology, risk factors, diagnosis, management and outcome. Acta Biomed.

[10] Zhang J, Chen Z, Zheng J, Breusch SJ, Tian J (2015). Risk factors for venous thromboembolism after total hip and total knee arthroplasty: a meta-analysis. Arch Orthop Trauma Surg.

[11] Malahias MA, Jang SJ, Gu A, Richardson SS, Chen AZ, Rao RD, Sculco PK (2021). Cervical spine degenerative disease is an independent risk factor for increased revision rate following total knee arthroplasty. Eur J Orthop Surg Traumatol.

[12] Lizaur-Utrilla A, Martinez-Mendez D, Collados-Maestre I, Marco-Gómez L, Lopez-Prats FA (2016). Elective total knee arthroplasty in patients with end-stage renal disease: is it a safe procedure?. J Arthroplast.

[13] Malkani JA, Heimroth JC, Ong KL, Wilson H, Price M, Piuzzi NS, Mont MA (2020). Complications and readmission incidence following total hip arthroplasty in patients who have end-stage renal failure. J Arthroplast.

[14] Lo IN, Tsai SW, Wu PK, Chen CF, Chang MC, Chen WM (2019). The mid-term outcome of dialysis-dependent patients undergoing primary total knee arthroplasty and total hip arthroplasty: a retrospective study. J Chin Med Assoc.

[15] Jhamb M, Weisbord SD, Steel JL, Unruh M (2008). Fatigue in patients receiving maintenance dialysis: a review of definitions, measures, and contributing factors. Am J Kidney Dis.

[16] Johansen KL, Kaysen GA, Dalrymple LS, Grimes BA, Glidden DV, Anand S, Chertow GM (2013). Association of physical activity with survival among ambulatory patients on dialysis: the comprehensive dialysis study. Clin J Am Soc Nephrol.

[17] Nanovic L (2005). Electrolytes and fluid management in hemodialysis and peritoneal dialysis. Nutr Clin Pract.

[18] Charra B, Calemard E, Uzan M, Terrat JC, Vanel T, Laurent G (1985). Carpal tunnel syndrome, shoulder pain and amyloid deposits in long-term haemodialysis patients. Proc Eur Dial Transplant Assoc Eur Ren Assoc.

[19] Ottesen TD, Zogg CK, Haynes MS, Malpani R, Bellamkonda KS, Grauer JN (2018). Dialysis patients undergoing total knee arthroplasty have significantly increased odds of perioperative adverse events independent of demographic and comorbidity factors. J Arthroplast.

[20] Neild GH (2017). Life expectancy with chronic kidney disease: an educational review. Pediatr Nephrol.

[21] Chang CH, Hsieh PH, Cashman J, Goyal N, Parvizi J (2015). Total hip arthroplasty in patients undergoing dialysis. The hip: preservation, replacement, and revision, 1st ed.

[22] Al-Jaishi AA, Oliver MJ, Thomas SM, Lok CE, Zhang JC, Garg AX, Kosa SD, Quinn RR, Moist LM (2014). Patency rates of the arteriovenous fistula for hemodialysis: a systematic review and meta-analysis. Am J Kidney Dis.

[23] Sridharan ND, Fish L, Yu L, Weisbord S, Jhamb M, Makaroun MS, You TH (2018). The associations of hemodialysis access type and access satisfaction with health-related quality of life. J Vasc Surg.

[24] Warren DJ, Otieno LS (1975). Carpal tunnel syndrome in patients on intermittent haemodialysis. Postgrad Med J.

[25] Paiva Filho HR, Pedroso FLC, Bueno FB, Paiva VGN, Oliveira EF, Rocha MA (2020). Prevalence of anxiety and depression symptoms in people with carpal tunnel syndrome. Rev Bras Ortop (Sao Paulo).

[26] Pan X, Wang J, Lin Z, Dai W, Shi Z (2019). Depression and anxiety are risk factors for postoperative pain-related symptoms and complications in patients undergoing primary total knee arthroplasty in the United States. J Arthroplast.

[27] London GM, Pannier B, Marchais SJ, Guerin AP (2000). Calcification of the aortic valve in the dialyzed patient. J Am Soc Nephrol.

[28] Moe SM, Chen NX (2008). Mechanisms of vascular calcification in chronic kidney disease. J Am Soc Nephrol.

[29] Razzaque MS (2011). The dualistic role of vitamin D in vascular calcifications. Kidney Int.

[30] Taniguchi T, Morimoto T, Shiomi H, Ando K, Kanamori N, Murata K, Kitai T, Kawase Y, Izumi C, Kato T, Ishii K, Nagao K, Nakagawa Y, Toyofuku M, Saito N, Minatoya K, Kimura T, CURRENT AS registry investigators (2018). Sudden death in patients with severe aortic stenosis: observations from the CURRENT AS Registry. J Am Heart Assoc.

[31] Nitta K, Yajima A, Tsuchiya K (2017). Management of osteoporosis in chronic kidney disease. Intern Med.

[32] United States Renal Data System. Annual Data Report 2017, Vol.2, Chapter 11. https://www.usrds.org/adr.aspx. Accessed 4 August 2021.

[33] Robinson BM, Bieber B, Pisoni RL, Port FK (2012). Dialysis outcomes and practice patterns study (DOPPS): its strengths, limitations, and role in informing practices and policies. Clin J Am Soc Nephrol.

